# *L. pneumophila* resists its self-harming metabolite HGA via secreted factors and collective peroxide scavenging

**DOI:** 10.1128/mbio.01207-23

**Published:** 2023-09-20

**Authors:** Mische Holland, Danielle N. Farinella, Emily Cruz-Lorenzo, Madelyn I. Laubscher, Darian A. Doakes, Maria A. Ramos, Nanami Kubota, Tera C. Levin

**Affiliations:** 1 Department of Biological Sciences, University of Pittsburgh, Pittsburgh, Pennsylvania, USA; 2 Department of Plant and Microbial Biology, University of California Berkeley, Berkeley, California, USA; 3 Department of Microbiology and Molecular Genetics, University of Pittsburgh School of Medicine, Pittsburgh, Pennsylvania, USA; Florida International University, Miami, Florida, USA; University of Washington, Seattle, Washington, USA

**Keywords:** *Legionella pneumophila*, natural antimicrobial products, reactive oxygen species, antimicrobial tolerance, interbacterial antagonism, density-dependent responses, homogentisic acid

## Abstract

Many pathogenic bacteria, including *Legionella pneumophila*, infect humans from environmental reservoirs. To survive in these reservoirs, bacteria must withstand microbe-on-microbe competition. We previously discovered that *L. pneumophila* can compete with neighboring bacteria via an antimicrobial metabolite called homogentisic acid (HGA). Curiously, *L. pneumophila* strains that secrete HGA are not wholly immune to its effects: low-density bacteria are strongly inhibited by HGA, whereas high-density cells are tolerant. How do these bacteria tolerate HGA and avoid self-harm during interbacterial competition? Here, we find that HGA toxicity occurs via the production of toxic hydroperoxides, and multiple factors facilitate high-density tolerance. First, HGA becomes fully toxic only after >1 h of oxidation. While this manifests as a delay in killing within well-mixed liquid cultures, in a biofilm environment, this could provide time for HGA to diffuse away before becoming toxic. Second, HGA generates quantities of hydroperoxides that can be collectively scavenged by high-density, but not low-density, cells. Third, high-density cells produce one or more secreted factors that are transiently protective from HGA. In combination, we propose that the bacteria are able to deploy HGA to generate a pool of reactive oxygen species surrounding their own biofilms while maintaining non-toxic conditions within them. Overall, these findings help to explain how broadly toxic molecules can be used as inter-bacterial weapons. They also provide insights about why some of our current decontamination methods to control *L. pneumophila* are ineffective, leading to recurrent disease outbreaks.

**IMPORTANCE:**

Before environmental opportunistic pathogens can infect humans, they must first successfully grow and compete with other microbes in nature, often via secreted antimicrobials. We previously discovered that the bacterium *Legionella pneumophila*, the causative agent of Legionnaires’ disease, can compete with other microbes via a secreted molecule called HGA. Curiously, *L. pneumophila* strains that produce HGA is not wholly immune to its toxicity, making it a mystery how these bacteria can withstand the “friendly fire” of potentially self-targeting antimicrobials during inter-bacterial battles. Here, we identify several strategies that allow the high-density bacterial populations that secrete HGA to tolerate its effects. Our study clarifies how HGA works. It also points to some explanations of why it is difficult to disinfect *L. pneumophila* from the built environment and prevent disease outbreaks.

## INTRODUCTION

As bacteria compete for nutrients and space, many species use antimicrobial compounds to antagonize their neighbors (1). While some of these molecules target only specific strains, those that generate reactive oxygen species (ROS) tend to be broadly active, as ROS can damage DNA, proteins, lipids, and other biological molecules (2). In oxygenated environments, redox-active secreted compounds, such as phenazines, quinones, and related molecules, can generate toxic ROS through redox cycling (3). Yet, despite their toxicity, many bacteria secrete these molecules in high quantities, suggesting that redox-active compounds can confer a number of benefits on the bacteria that produce them, such as improved cellular respiration and improved scavenging of limiting nutrients from the environment (4 – 6).

*Legionella pneumophila* is an aquatic opportunistic human pathogen that can cause large disease outbreaks if it colonizes building plumbing systems (7). We previously described the secreted quinone homogentisic acid (HGA) as an antimicrobial metabolite used by *Legionella pneumophila* (8). In stationary phase when cells are a high density, this bacterium secretes abundant (50–500 µM) HGA, which can spread across an agar plate to kill neighboring *Legionella* species. We demonstrated that HGA’s oxidation state was relevant for its activity, that reducing agents made HGA inhibition less potent, and that HGA was non-toxic in anaerobic conditions. We, therefore, proposed that reactive intermediates were inhibitory, although HGA’s mechanism of action was unknown. In addition, we previously observed that *L. pneumophila* could itself be antagonized by HGA under certain conditions. Unexpectedly, *Legionella* growth phase had no impact on HGA susceptibility or tolerance. Instead, susceptibility depended on cell density; dense cells (~2E9 CFU/mL) were insensitive to 24 h of HGA treatment, but the same strain was extremely sensitive when diluted (~2E7 CFU/mL) (8). Thus, in a spatially structured environment such as a biofilm, high-density cells that produce HGA are not susceptible to this molecule, but any neighboring, low-density cells outside the biofilm are at risk. HGA susceptibility was particularly strong when cells were exposed to HGA in phosphate-buffered saline (PBS), which more closely approximates the low-nutrient, aquatic conditions where *Legionella* spp. are found than rich media conditions. In summary, we found that *L. pnuemophila* strains that antagonize neighbors with secreted HGA can themselves be extremely sensitive to this same molecule. How, then, is *L. pneumophila* able to use HGA to kill other bacteria while avoiding self-harm?

Here, we investigate HGA’s mechanism of action and its density-dependent susceptibility. While HGA itself is non-toxic, our findings suggest that it spontaneously generates toxic hydroperoxides over time in aerobic conditions. While dilute *L. pneumophila* cells are highly sensitive, more dense bacteria can tolerate >1,000-fold higher HGA concentrations. Moreover, the threshold separating high-density tolerant cells and low-density susceptible cells is incredibly sharp, separated by only a 2×–3× difference in cell density. How is this switch-like difference in susceptibility achieved? First, we find that high-density cells secrete one or more factors (likely small molecule antioxidants) that reduce the amounts of toxic ROS generated by HGA; the secreted factor(s) can transiently protect low-density cells from HGA. Second, HGA generates concentrations of H_2_O_2_ that high-density cells can cooperatively detoxify but low-density cells cannot. Third, because HGA takes >1 h to begin to produce toxic levels of ROS in aerobic environments, we propose that this time delay acts as a metaphorical long fuse, allowing HGA to diffuse away from high-density cells in a biofilm before generating toxic ROS. Together, these dynamics permit high-density *L. pneumophila* to secrete abundant HGA without suffering any loss in viability.

## RESULTS

### HGA induces rapid, density-dependent cell death

**FIG 1:**
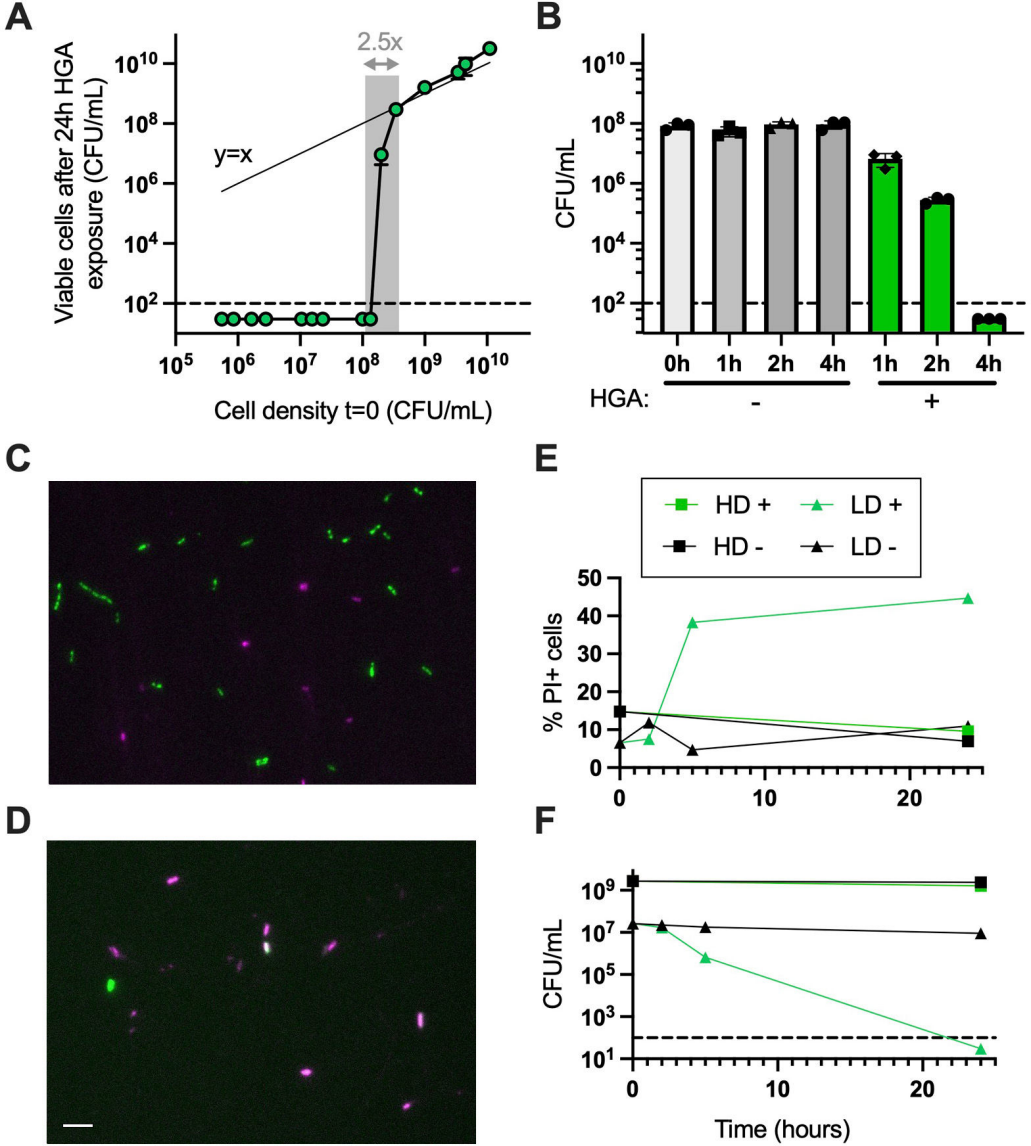
HGA induces rapid, density-dependent cell death. (**A**) At high densities, *L. pneumophila* cells are insensitive to HGA; CFUs recovered before and after HGA exposure are similar, matching the *y* = *x* line (thin line). However, within only a 2.5-fold change in starting cell density (shaded region), *Legionella* cells transition from fully tolerant of HGA to completely susceptible. (**B**) Low-density *Legionella* cells die rapidly from 125-µM HGA, with CFUs unrecoverable after 4 h. (**C and D**) Representative 24-h images of green fluorescent protein (GFP) positive *Legionella* exposed to HGA at high (**C**) or low density (**D**) and stained with PI (magenta) to visualize dead cells. Scale bar = 5 µM. (**E**) Quantification of PI+ cells and (F) viable CFU/mL during microscopy experiment of HD and LD cells incubated both with (+) and without (−) HGA. Dashed line shows the limit of detection in all experiments. HD, high density; LD, low density; PI, propidium iodide.

To precisely define when and how HGA inhibition occurs, we first investigated which cell densities were susceptible to HGA. When we exposed cells across a range of cell densities to 125-µM HGA for 24 h, we found that the transition between complete HGA susceptibility and full tolerance was incredibly sharp, spanning only a 2.5- to 3.0-fold difference in starting cell density (1.35E8 vs 3.45E8 CFU/mL, Fig. 1A). Above the density threshold, *Legionella* cells were essentially unaffected by HGA, as the number of CFUs at *t* = 0 matched the number we recovered at *t* = 24 h (*y* = *x* line, Fig. 1A). Below the threshold, we did not recover any viable CFUs. We interpret this switch-like behavior to indicate HGA sensitivity is a biologically regulated process, rather than a generic outcome resulting from more cells being present in high-density cultures.

We also found that HGA killed low-density (<7E7 CFU/mL) cells quickly. CFUs began to decline after 1 h, and full inhibition occurred as early as 4 h (Fig. 1B). The kinetics of inhibition varied, depending on culture volume and shaking speed (Fig. S1), likely due to the efficiency of oxygenation. To determine if HGA inhibition is bactericidal or if it is driving cells into a viable but non-culturable state, we exposed GFP-expressing *Legionella* to HGA and stained dead cells with propidium iodide (PI) (Fig. 1C and D). We found up to 45% of low-density cells exposed to HGA became PI+. Additionally, the proportion of PI+ cells increased at the same time that these cells experienced a drop in CFUs (Fig. 1E and F), indicating that the low-density *Legionella* cells are dying after exposure to HGA. Notably, our % PI+ measurements in this assay are likely an underestimate of total dead cells, as we expect to miss cells that have already lysed. Overall, we conclude that HGA is bactericidal and kills low-density cells rapidly. High-density cells remain unaffected, and there is a tight density threshold separating the two outcomes of dramatic cell death vs complete HGA tolerance.

### Density-dependent susceptibility to HGA and H_2_O_2_

**FIG 2:**
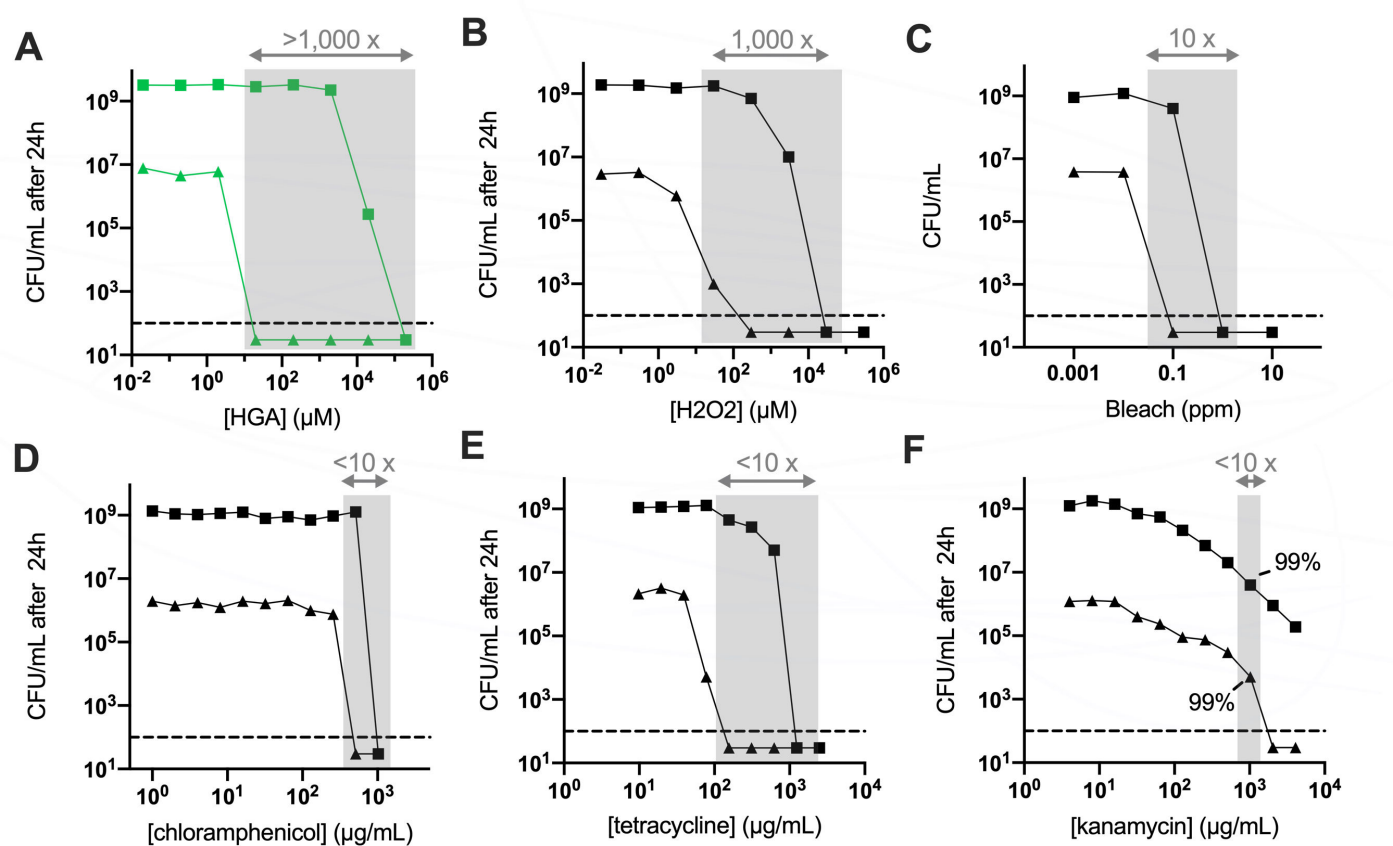
*L. pneumophila* cell density has a large impact on HGA and H_2_O_2_ susceptibility. (**A**) HGA kills low-density bacteria (triangles) at ~5,000× lower concentration than that required to kill high-density cells (squares) after 24 h. Shaded regions show the differences in inhibitory concentrations between high- and low-density *Legionella* for each compound.** (B and C**) Density-dependence of *Legionella* susceptibility to H_2_O_2_ (**B**) and, to a lesser extent, bleach (**C**). **(D through F**) *Legionella* susceptibility to various antibiotics shows a more typical inoculum effect. In panel F, where solubility issues limited our ability to kill high-density cells below our limit of detection, we highlight the amount of drug required to kill 99% of the starting bacterial population. All experiments incubated bacteria with antimicrobial compounds in PBS for 24 h.

Many antibiotics are known to produce an “inoculum effect,” wherein higher antibiotic concentrations are required to kill dense bacteria as compared to dilute bacteria (9, 10). To determine if HGA’s density dependence behaved similarly to inoculum effects, we measured the minimum bactericidal concentration (MBC) for high- and low-density *Legionella* exposed to a variety of antimicrobial compounds in minimal PBS media. Strikingly, over 1,000-fold more HGA was required to fully kill high-density cells (20-mM HGA) than low-density cells (20-µM HGA, Fig. 2A). We observed a similar density dependence in *Legionella*’s sensitivity to H_2_O_2_ (Fig. 2B, 30 µM vs 30 mM to kill >99.9% of low- vs high-density cells).

In contrast to this density-linked sensitivity, we observed that *L. pneumophila* treated with bleach, multiple antibiotics, and the toxic lipid 4-HNE, exhibited at most a 10-fold difference in the MBCs between high- and low-density *L. pneumophila* (Fig. 2C through F; Fig. S2A). As an additional oxidative stress, we tested *Legionella*’s susceptibility to paraquat, a chemical that generates superoxide. Although previous research found *L. pneumophila* was sensitive to 0.25- to 0.5-mM paraquat in rich media (11, 12), in the minimal media used here, we found *L. pneumophila* tolerated up to 21-mM paraquat with minimal cell death at both high and low cell densities (Fig. S2). We note that the MBCs measured here were higher than previously reported MBCs and minimum inhibitory concentrations (MICs) for *Legionella pneumophila* (13 – 15). This discrepancy likely results from the fact that antimicrobials are typically added during bacterial log phase growth in chemically complex, rich media, while our treatments are added to non-replicative cultures in minimal media. For HGA, we previously showed that this compound is more toxic in minimal vs rich media, and this difference is due to high concentrations of cysteine in rich media, which acts as an antioxidant to protect from HGA (8). Overall, *L. pneumophila*’s density-dependent susceptibility to both HGA and H_2_O_2_ is several orders of magnitude stronger than the inoculum effect we observed for other antimicrobial compounds (Fig. 2A and B vs Fig. 2C through F ). When we tested several other species of bacteria (*Legionella micdadei*, *Pseudomonas fluorescens*, *Klebsiella aerogenes*, and *Bacillus subtilis*) in the same assay, *L. micdadei* and *B. subtilis* were both susceptible to HGA. However, only *L. pneumophila* exhibited a strong density dependence to both HGA and H_2_O_2_ at the concentrations tested (Fig. S2).

### HGA tolerance is independent of the Lqs quorum sensing and HGA synthesis pathways

At high density, bacteria often experience nutrient limitation, slowed growth, and other stresses that could alter bacteria sensitivity to antimicrobials. However, we previously found that *L. pneumophila*’s high-density tolerance of HGA was neither affected by log vs stationary growth phase nor impacted by the stringent response pathway, which coordinates many stress responses in high-density, stationary phase bacteria (8, 16). Instead, HGA susceptibility was linked specifically to cell density. Therefore, we next tested whether *Legionella* regulates its density-dependent responses to HGA via candidate quorum sensing pathways. During quorum sensing, bacteria sense their own cell density through the secretion of autoinducer molecules, which accumulate to high concentrations when cells are dense and activate downstream signaling pathways (17, 18). Within the sole characterized *Legionella* quorum sensing (Lqs) pathway in *L. pneumophila*, the protein LqsA synthesizes a secreted autoinducer, which is sensed by transmembrane kinases, ultimately altering the activity of the response regulator LqsR (19). We previously found that wild-type cells and a ∆*lqsR* mutant had similar HGA susceptibility (8), suggesting that this protein is not involved in regulating HGA susceptibility. However, a second response regulator, LvbR, was recently discovered in the Lqs pathway, with some downstream target genes that are not regulated by LqsR (20). Signaling by LvbR, but not LqsR, was also found to be important for *Legionella* development into antibiotic tolerant persister cells (21). To test if LvbR or another part of the Lqs pathway regulates HGA tolerance, we constructed deletion mutants of *lvbR* and *lqsA*. We found that all Lqs mutants had similar HGA susceptibility to wild type (Fig. S3), supporting and strengthening our prior conclusions that density-dependent HGA susceptibility is regulated by a mechanism independent of the Lqs pathway.

One secreted molecule that accumulates specifically in high-density *Legionella* cultures is HGA itself. We next considered the possibility that HGA could function as an autoinducer, as secreted quinones have been shown to act as signaling molecules in other bacterial species (22). In this scenario, high-density cells could begin to secrete HGA, sense the initial, relatively low concentrations of extracellular HGA around them, and activate their protective downstream pathways before releasing subsequent toxic levels of HGA. If this hypothesis is correct, we predicted that *Legionella* mutants that are unable to secrete HGA would be susceptible to exogenous HGA, even at high cell density. Contrary to this expectation, we observed that a mutant that secretes no HGA (Tn:*hisC2* [8]) showed similar patterns of susceptibility to exogenous HGA as both wild-type cells and ∆*hmgA* mutants that secrete excess HGA due to an inability to recycle HGA back into central metabolism (Fig. S3). We, therefore, concluded that *Legionella*’s density-dependent susceptibility is unrelated to both HGA biosynthesis and Lqs pathway signaling.

### *L. pneumophila* RNA-seq reflects low-density cell oxidative stress from HGA

To identify genes that could underlie *Legionella*’s density-dependent susceptibility to HGA, we compared the transcriptomes of high- and low-density cells, both with and without HGA exposure (i.e., high density [HD]+, HD−, low density [LD]+, and LD− conditions). Because LD+ cells begin to experience substantial cell death after 1–2 h of HGA exposure (Fig. 1B), we analyzed RNA from each condition at the *t* = 1-h time point. By principal components analysis, the transcriptional profiles from HD samples were clearly separated from LD samples along PC1, while LD− and LD+ samples differed along PC2 (Fig. S4). The LD+ samples also had the most within-condition variation. Because LD+ cells experience a steep decline in CFUs between *t* = 0 h and *t* = 2 h, we expect that the differences among replicates reflect slight experimental variations in timing or degree of ROS generation from HGA. In agreement with the PCA, correlation matrices of the normalized read counts within conditions showed low variation between replicates within each condition except for the LD+ condition (Fig. S4).

From the RNA-seq data, we first asked whether high-density cells produced an inducible, protective response to HGA by comparing HD− and HD+ samples. Contrary to this hypothesis, we found that zero genes were differentially expressed when high-density cells were exposed to 125 µM HGA (Table S1). Thus, in addition to high-density cells’ HGA tolerance (i.e., they have no decline in CFUs, Fig. 1B), these cells do not appear to activate any stress or signaling responses to HGA at the transcriptional level.

**FIG 3:**
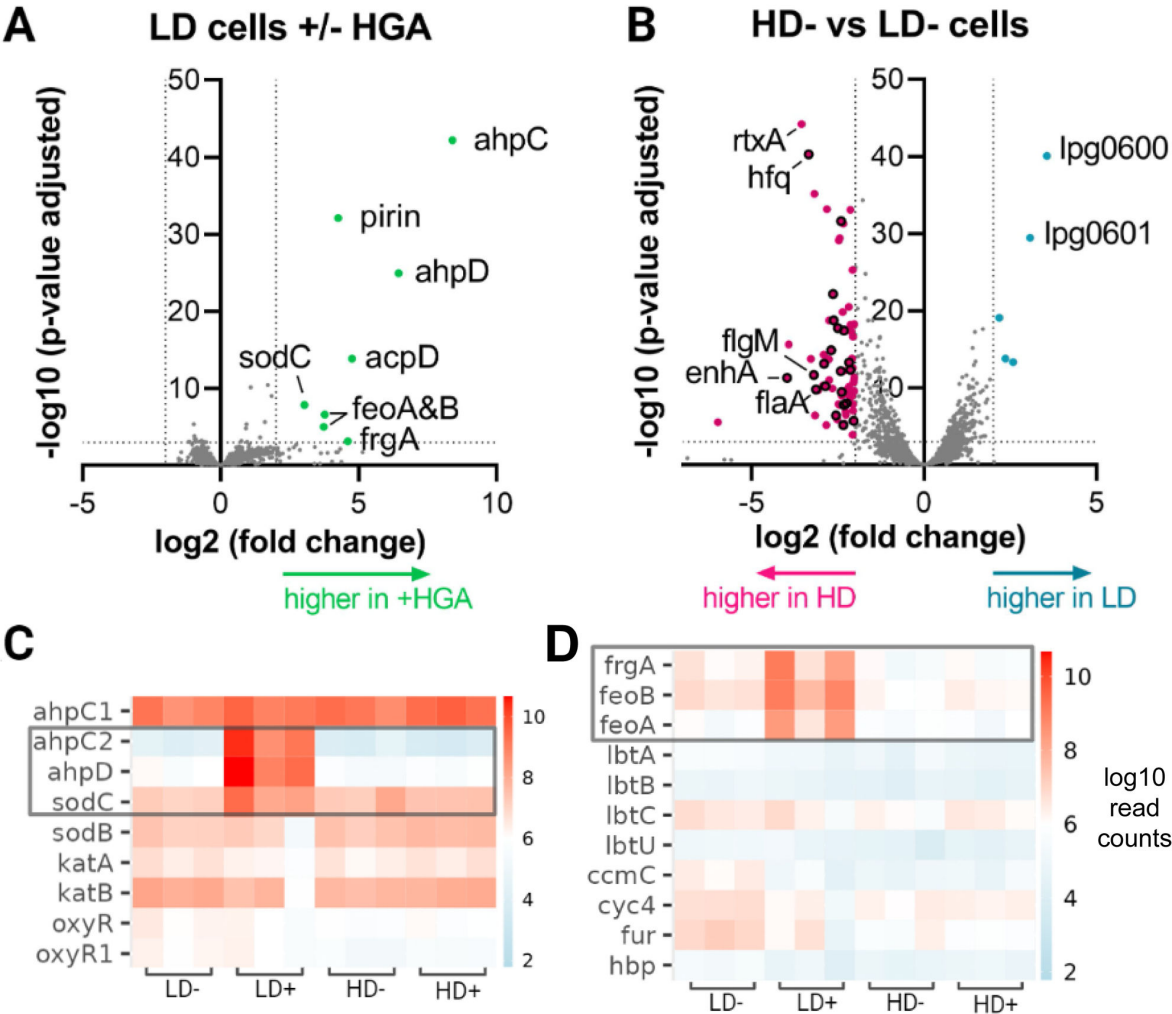
Differentially expressed genes identified by RNA-seq. (A) Volcano plot showing differential gene expression in low-density *L. pneumophila* with and without HGA exposure. Dotted lines indicate significance thresholds and significantly different genes are colored. (B) Differential expression between high-density and low-density cells in the absence of HGA. Genes related to flagellar function are outlined in black. (C and D) Heatmaps of RNA expression level for key genes in the oxidative stress (C) and iron scavenging pathways (D). Each condition examined bacteria at high or low cell density (HD vs LD), either with or without HGA (+/−). Gray box indicates genes that were significantly upregulated in LD+ cells.

In contrast, low-density cells robustly upregulated eight genes when exposed to HGA, most related to oxidative stress (Fig. 3A). These included *sodC* (*lpg2348*), a copper-zinc superoxide dismutase, and *ahpC2D* (*lpg2349* and *2350*), which encodes an alkyl hydroperoxidase and reductase that together scavenge hydroperoxides such as H_2_O_2_ (12). Also upregulated was an operon containing *pirin* and *acpD* orthologs (*lpg2286* and *2287*). While both genes are uncharacterized in *Legionella*, pirins are found across bacteria and eukaryotes where they play diverse roles, including acting as redox-sensing transcriptional regulators (23) or directly degrading aromatic antimicrobial compounds (24). AcpD is an azoreductase, acting on aromatic nitrogen-containing compounds (25). The final three upregulated genes were related to iron acquisition. The *feoAB* operon (*lpg2658*/*2657*) mediates the transport and uptake of Fe^2+^, while *frgA* (*lpg2800*) synthesizes a siderophore for uptake of Fe^3+^ (26). Oxidative stress has previously been shown to induce an iron starvation response via oxidation of Fe^2+^ and subsequent release of the Fur repressor (27), consistent with the gene expression observed here. Notably, the eight genes upregulated in LD+ conditions represent only a subset of *Legionella*’s oxidative stress response genes. In none of the RNA-seq conditions did we observe differential expression of *Legionella*’s other superoxide dismutase *sodB*, nor the other alkyl hydroperoxidase *ahpC1*, nor any previously characterized catalase or *oxyR* regulator genes (Fig. 3CFig. 3; Table S1). Similarly, none of the *lqs* pathway genes nor the HGA biosynthesis genes were differentially expressed (Fig. S4).

Because high-density *Legionella* spp. show no change in viability or gene expression when exposed to HGA, we hypothesized that the mechanisms of high-density tolerance could be due to differences between high- and low-density cells prior to HGA exposure. Between HD− and LD− conditions, there were 78 genes significantly upregulated in high-density cells and 5 genes upregulated in low-density cells. *Legionella* exhibits a biphasic lifestyle and is known to upregulate flagellar biosynthesis and virulence gene expression in stationary phase, downstream of the stringent response pathway (28, 29). The predominant transcriptional profile of genes upregulated in HD− cells was consistent with this flagellar regulon activation. Twenty-one of the 78 upregulated HD− genes were flagellar (in flagellar gene classes II, III, and IV) or previously characterized as part of the *fliA* flagellar regulon (28). If we relax our significance threshold to *P* < 0.05, 60% of all flagellar-related genes in the genome (34 of 57) were upregulated in HD− as compared to LD− conditions (Table S1). Relatedly, nine characterized virulence genes (i.e., toxins or effectors) were upregulated in HD− conditions. While this was the major transcriptional signature, we previously showed that HGA susceptibility was unaffected by mutations to the stringent response pathway (8), which are expected to perturb activation of the flagellar regulon (29). Therefore, we predict that HGA tolerance is more likely to be conferred by one or more of the remaining, non-flagellar genes upregulated in high-density cells. Of these, there was no obvious pattern, partly due to the fact 47% of the remaining genes were hypothetical and uncharacterized, mostly small proteins that are predicted to be unstructured by AlphaFold. There were also two heat shock proteins belonging to the HSP20 family (*lpg2191* and *2493*) as well as several predicted transcriptional regulators that could mediate the expression of genes at high density (e.g., *lpg0476*, *0586*, *2008*, *2181*, and *2145*).

Within low-density cells, the two most upregulated were *lpg0600*, an Rrf2 family transcription factor, and *lpg0601*, an ABC transporter. Notably, these genes lie closely upstream of *lpg0607*, a gene that was repeatedly disrupted in a screen for HGA-tolerant mutants in our previous study (8), although *lpg0607* itself was only weakly upregulated in low-density cells (1.8-fold higher, *P* = 0.043). It is possible that regulation of this operon modulates HGA sensitivity in low-density bacteria. Overall, the RNA-seq results revealed many genes differentially regulated between high- and low-density cells, as well as specific oxidative stress responses in HGA-exposed, low-density *Legionella*.

### HGA gradually produces hydroperoxides, which can be quenched by high-density cells

**FIG 4:**
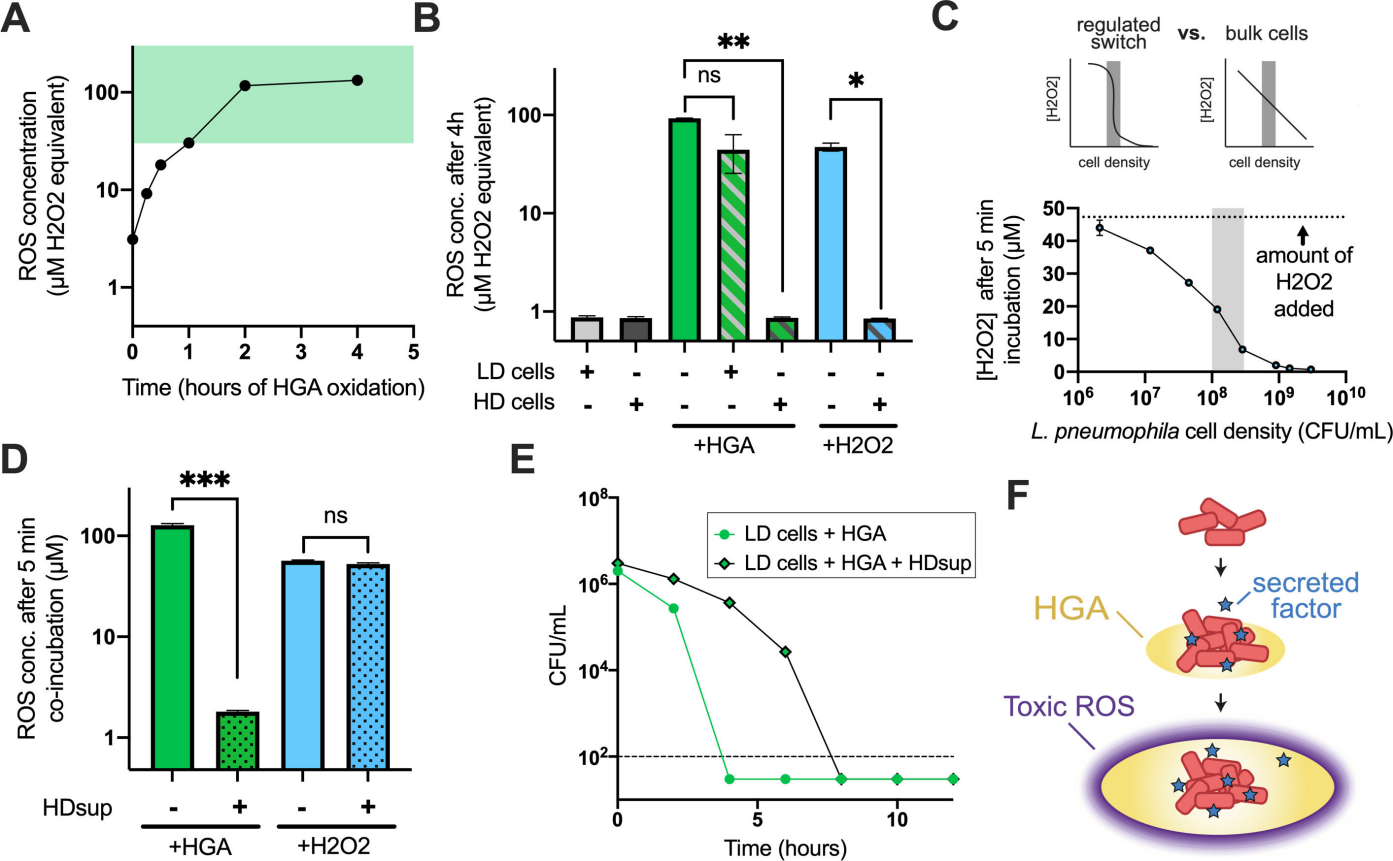
Dynamics of HGA toxicity, collective ROS detoxification, and secreted anti-ROS factors allow high-density cells to survive HGA exposure. (**A**) Generation of ROS upon oxidation of 125-µM HGA, measured with Amplex Red. Green-shaded region marks H_2_O_2_ concentrations that were toxic to low-density, but not high-density, cells in Fig. 2. (**B**) HD *L. pneumophila* spp. reduce the amount of ROS present in HGA- or H_2_O_2_-treated cultures, but LD cells do not. *P < 0.05, **P < 0.005 by Welch’s *t*-test. (**C**) To test if the reduction in H_2_O_2_ in the presence of HD cells in panel B was due to the bulk action of many cells vs a density-dependent switch as we saw for HGA, *L. pneumophila* of many cell densities were incubated in the presence of 50-µM H_2_O_2_ for 5 min, then the remaining H_2_O_2_ was quantified with Amplex Red. The reduction in H_2_O_2_ was correlated with cell density but did not exhibit regulated switch behavior. Shaded region shows the cell density threshold observed for HGA in Fig. 1A. (**D**) Amplex Red measurements of ROS produced *in vitro* by H_2_O_2_ or partially oxidized HGA. After 5 min, co-incubation with supernatants from high-density *L. pneumophila* (HDsup), the Amplex Red signal from HGA was significantly reduced (*P* < 0.0005), but signal from H_2_O_2_ was unaffected. (**E**) Incubation of low-density cells with HDsup provides transient protection from HGA. (**F**) Proposed model of HGA activity in biofilms. HGA is secreted by high-density cells and only forms toxic levels of ROS after a time delay and spread of HGA. Toxic ROS levels near the high-density cells are kept low via both cooperative ROS detoxification and secreted factors that transiently quench ROS production from HGA. ns, not significant.

Because H_2_O_2_ and HGA displayed a strikingly similar density dependence in *L. pneumophila* (Fig. 2A and B) and because *ahpC2D*, genes used to detoxify hydroperoxides, were highly upregulated in LD+ cells (Fig. 3A), we next hypothesized that HGA kills low-density *L. pneumophila* via generation of H_2_O_2_ or a related hydroperoxide. To test this hypothesis, we used the reagent Amplex Red, which generates the fluorescent compound resorufin in the presence of hydroperoxides (30). Previously, we found that HGA was a non-toxic in anaerobic conditions (8), suggesting that toxic compounds are produced upon oxidation. Consistent with this finding and with the timing of HGA killing of low-density cells (Fig. 1B), we saw that synthetic HGA incubated in shaken test tubes gradually produced an abundant fluorescent signal. When compared to a standard curve of H_2_O_2_, 125 µM HGA generated an equivalent signal of 130 µM H_2_O_2_, reaching hydroperoxide levels that are toxic to low-density cells after 1 h (Fig. 2 and Fig. 4). As we found that high-density cells could tolerate H_2_O_2_ but low-density cells died between 30 and 3,000 µM H_2_O_2_ (Fig. 2B), the fact that HGA generates hydroperoxides within this critical range may contribute to *L. pneumophila*’s density-dependent susceptibility to HGA.

However, it is also possible that HGA generates different types or concentrations of reactive oxygen species in the presence of bacteria. To test this hypothesis, we incubated HGA alone or in combination with *L. pneumophila* at high or low density for 4 h and measured hydroperoxide concentrations with Amplex Red. We saw that HGA alone generated the equivalent of 92-µM H_2_O_2_ and only a slightly lower amount (52 µM) in the presence of low-density cells. However, when HGA was incubated with high-density *L. pneumophila*, the Amplex Red signal was dramatically reduced (0.8 µM, Fig. 4). This behavior was not unique to HGA, as high-density cells also depleted H_2_O_2_ during co-incubation (Fig. 4B).

H_2_O_2_ is cell permeable, and it has previously been shown that the combined actions of bacteria scavenging intracellular H_2_O_2_ can collectively reduce local H_2_O_2_ concentrations (31, 32). Because cells at higher density should be better at scavenging H_2_O_2_, we next investigated whether this phenomenon was sufficient to explain the density-dependent susceptibility to HGA. If so, we predicted that there would be a switch-like transition between 1E8 and 3E8 CFU/mL (the density threshold defined in Fig. 1A), where cultures above this threshold would scavenge H_2_O_2_ much more efficiently than bacteria below the threshold (see “regulated switch”; Fig. 4). Alternatively, if high-density H_2_O_2_ scavenging is simply related to the activity of additional cells, we predicted that cells at higher density would scavenge H_2_O_2_ without any apparent density threshold (see “bulk cells”; Fig. 4). When we briefly incubated cells at different densities with 50 µM H_2_O_2_, we observed that higher cell densities reduced the concentration of H_2_O_2_ more readily, but this occurred without any apparent density threshold (Fig. 4C). Therefore, while high-density cells exhibit enhanced H_2_O_2_ scavenging as expected, this phenomenon is not sufficient to explain HGA’s density-dependent susceptibility.

### High-density cells secrete a transiently protective molecule

We next asked whether the high-density cells’ quenching activity could be enhanced by a secreted factor. We generated secreted supernatants from high-density cells (HDsup) by incubating dense *L. pneumophila* in PBS for 1 h and then centrifuging and filter sterilizing the sample through a 0.2-µm filter to remove bacteria. To generate HGA-derived ROS, we oxidized HGA alone for 4 h. When we then incubated the high-density supernatants with HGA, we found that the HDsup could largely quench the Amplex Red signal (Fig. 4D). Unlike the activity from live cells, HDsup incubated with H_2_O_2_ did not alter the Amplex Red measurement. This suggests that high-density cells rapidly secrete one or more factors that are protective from HGA. Because HDsup does not substantially alter H_2_O_2_ readings, we infer that the decline in fluorescence is due to HGA-derived ROS quenching and that HDsup does not interfere with the Amplex Red assay. Moreover, because HDsup does not detoxify H_2_O_2_ but does alter ROS generated from HGA, we conclude that HGA produces one or more toxic ROS that react with the Amplex Red reagent but are distinct from H_2_O_2_.

If high-density cells resist HGA via a secreted factor, we predicted that HDsup would protect low-density cells from HGA. Indeed, we found that low-density cells incubated with HDsup experienced delayed cell death from HGA, extending the time to death by at least 4 h (Fig. 4D). This effect was transient, and the cells still died by *t* = 8 h. Nevertheless, these experiments reveal that HGA generates quantities of ROS that are toxic to low-density cells, but that high-density cells can transiently quench or scavenge extracellular HGA-derived ROS via a secreted, protective factor.

## DISCUSSION

We previously discovered that *L. pneumophila* can use HGA as a secreted, antimicrobial metabolite to antagonize neighboring *Legionella* bacteria (8). Here, we have shown that HGA is bactericidal, clarified its mechanism of action, and uncovered mechanisms that mediate *L. pneumophila*’s unusual, density-dependent susceptibility to this molecule. We find that low-density *L. pneumophila* cells are over 1,000× more sensitive to HGA than high-density bacteria (Fig. 2). This sensitivity is mirrored in *L. pneumophila*’s responses to H_2_O_2_ but is distinct from other antimicrobials or oxidative stressors (Fig. 2; Fig. S2). Based on these observations, as well as the upregulation of alkyl hydroperoxidases in LD+ samples (Fig. 3A), and the measurement of HGA-derived ROS using Amplex Red (Fig. 4C), we propose that HGA generates toxic hydroperoxides (distinct from H_2_O_2_), which kill low-density *L. pneumophila*. Supporting this conclusion, we found that *L. micdadei* and *B. subtilis* are sensitive to HGA but relatively insensitive to H_2_O_2_ (Fig. S2). Further ruling out H_2_O_2_ as an intermediate, *L. micdadei tatlock* is also a naturally catalase-positive strain (33), yet it remains susceptible to HGA and we found previously that catalase was not protective against HGA (8). While Amplex Red is specific for hydrogen peroxide over superoxide, other compounds including peroxynitrite (34) and some organic hydroperoxides (35) have previously been found to react in the Amplex Red/horseradish peroxidase assay, which could mediate HGA toxicity.

We also found that HGA susceptibility in *L. pneumophila* follows an extremely sharp density threshold; the only difference between bacteria that are fully impervious to HGA and those that are completely susceptible is a two- to threefold difference in starting cell density (Fig. 1). We interpret this sharp transition to be the result of active, density-dependent regulation. So, what explains this switch-like difference in HGA susceptibility in *L. pneumophila*? Our findings show that this phenomenon is distinct from the “inoculum effect” typically seen for antibiotics (Fig. 2), the HGA biosynthesis pathway, and the sole, known quorum sensing pathway of *Legionella* (Lqs) (Fig. S3).

Instead, our results suggest that at least three phenomena contribute to high-density HGA tolerance. First, and best characterized previously, the combined actions of bacteria scavenging toxic hydroperoxides within their own cells can collectively reduce environmental peroxide concentrations, and this process occurs more quickly when more bacterial cells are present (31, 32). We find that HGA produces hydroperoxide concentrations that can be quickly, collectively scavenged by high-density but not low-density *L. pneumophila* populations (Fig. 4A through C). However, this collective scavenging alone is not sufficient to explain HGA’s density-dependent susceptibility.

Second, high-density *L. pneumophila* cells secrete unknown factors that partially quench HGA-derived ROS (Fig. 4D) and provide transient protection from HGA (Fig. 4E). This transient effect was not detectable in our prior study (8) because those experiments only tested samples after 24 h of HGA treatment. We hypothesize that the transient protection arises because the protective factor is degraded, used up, or oxidized as it interacts with ROS. However, if high-density cells continue to secrete fresh protective factors, these molecules could provide longer-lasting protection.

Third, we propose that the time delay between HGA secretion and the production of toxic ROS could provide an additional level of protection in spatially structured environments. We initially discovered that HGA could kill bacteria in an agar plate assay wherein HGA produced by a *L. pneumophila* biofilm spread across the agar and killed neighboring bacteria (8). Because we found HGA is non-toxic when anaerobic (8), the HGA would likely remain non-toxic at the center of the producing biofilm, only beginning to oxidize and produce ROS once it reached the biofilm periphery. Even in highly oxygenated environments, we found that it takes >1 h for HGA to generate toxic levels of hydroperoxides (Fig. 4A), during which time HGA would continue to diffuse away from the high-density, HGA-producing cells. This delay in HGA toxicity, combined with local ROS scavenging and quenching near high-density cells, could create a low ROS zone near the site of secretion and abundant ROS at more of a distance. In this “toxic moat” model, ROS would surround, but not directly impact, high-density HGA-producing cells (Fig. 4F).

What is the protective factor that is secreted from high-density cells? Typically, bacteria counteract high concentrations of hydroperoxides with catalase enzymes and lower concentrations with peroxidases (36). We observed that low-density cells upregulated *ahpC2D* peroxidases upon HGA exposure (Fig. 3A). However, as neither peroxidases, catalases, nor any other typical ROS detoxifying protein was differentially regulated in HD− vs LD− cells (Fig. 3C), we expect these enzymes are unlikely to be responsible for the protective activity in high-density supernatants. Other secreted factors such as antioxidants could be responsible instead. Indeed, as we previously saw that reducing agents such as cysteine, glutathione, and dithiothreitol (DTT) were protective from HGA (8), similar molecules could provide the transient protective activity we observe in high-density supernatants.

Finally, these findings have potential relevance for public health. Because *L. pneumophila* within the built environment can cause serious disease outbreaks, methods of disinfection are of primary interest (37, 38). Many of the most common methods used, such as chlorine, chloramine, hydrogen peroxide, and ozone, act through the induction of oxidative damage. Our results suggest that *L. pneumophila* susceptibility to certain oxidative stressors strongly depends on cell density, particularly in low-nutrient, aquatic environments. Future decontamination approaches should take into account the fact that high-density bacteria (e.g., which may be found in biofilms) may be many orders of magnitude less susceptible to oxidizing agents than the low-density bacteria typically tested in the lab. For example, some decontamination efforts have used hydrogen peroxide well below the 30 mM concentration we find is necessary to fully kill high-density cell populations (38). High-density *L. pneumophila* that are insensitive to disinfection methods could form a persistent subpopulation that would be poised to recolonize water systems and contribute to the recurrent disease outbreaks caused by this pathogen (39).

## MATERIALS AND METHODS

### Strains and mutant construction

As in Levin et al., our wild-type *Legionella pneumophila* (*Lp*) strain was KS79, and mutants were constructed in this genetic background by allelic exchange using pLAW344. Briefly, we cloned these regions into pLAW344 via Gibson assembly, then transformed into *Lp*, selected for plasmid integration on buffered charcoal yeast extract (BCYE)-chloramphenicol plates, and counter-selected to create the knock out on BCYE-sucrose plates. To create the *lvbR* knockout strain, the 5′ genomic region was cloned using primers TL180_LvbR_P1_oH1 (acggtatcgataagcttga gcgtgctgattggtcc) and TL181_LvbR_P2_oH1 (ggtttatgaggctga ttgttttttcattc), while the 3′ region was cloned using TL182_LvbR_P3_oH2 (gaatgaaaaaacaa tcagcctcataaacc) and TL183_LvbR_P4_oH2 (cgctctagaactagtggatc cgcagtgccagtcatgac). We used an identical protocol to create the *lqsA* mutant, cloning the 5′ region with primers TL186_LqsA_P1_oH1 (acggtatcgataagcttga aatcccctgctccccaaaatag) and TL187_LqsA_P2_oH1 (cgccaataattagaagtcgtg ccataactcagattcttttcc) and cloning the 3′ region with primers TL188_LqsA_P3_oH2 (ggaaaagaatctgagttatgg cacgacttctaattattggcg) and TL189_LqsA_P4_oH2 (cgctctagaactagtggatc atgcaacatttctcaatacc). The GFP-positive *Lp* in the PI microscopy assay were generated by transforming KS79 with the pON-GFP constitutive expression plasmid (40).

The other bacterial strains and species used were *L. micdadei* tatlock (41, 42), *Pseudomonas fluorescens* SBW25 (43), *Klebsiella aerogenes* DBS0305938 (Dicty Stock Center ID), and *Bacillus subtilis* IAI (WT strain 168 *trpC2*) (44). *L. micdadei* was cultured on BCYE plates, and liquid cultures were grown in AYE, the same culture conditions used for *L. pneumophila. P. fluorescens* and *B. subtilis* were cultured at 28°C and 37°C, respectively, on Luria broth (LB) plates and liquid cultures were grown in LB. *K. aerogenes* was cultured at 37°C on SM/5 plates, and liquid cultures were grown in SM media(45).

### HGA and other antimicrobial susceptibility assays

HGA susceptibility assays were performed in PBS as previously described. Briefly, our core assay was performed with washed, early stationary phase bacteria resuspended in 3 mL PBS at 3E9-1E10 CFU/mL for high-density conditions and 1E7-7E7 CFU/mL for low density conditions, ±125 µM HGA. Cells were plated onto BCYE after 0–24 h of incubation, and colonies on plates were counted after 2–3 days of 37°C incubation for CFU viability.

Density-dependent susceptibility to various ROS stresses and antibiotic drugs was determined using a standard MBC99 protocol (46). High- and low-density cells were mixed with drugs in 200 μL of 1× PBS and incubated at 37°C in a shaker. Each condition was plated immediately for CFUs on BCYE and plated again after 24 h at 37°C incubation. The MBC99 or minimum amount of drug required to kill ≥99% of the bacteria in the untreated controls was determined using CFU/mL counts from each drug condition. The antibiotics tested were kanamycin (4–4096 µg/mL, Thermo Fisher #BP906-5), chloramphenicol (1–1024 µg/mL, Thermo Fisher BP904-100), and tetracycline (9.76–2,500 µg/mL; Sigma #87128–25G). The ROS producers included H_2_O_2_ (0.03–300,000 µM; Fisher Scientific #H325), 4HNE (0.1–1,000 µM; Cayman Chemical #321001), and paraquat (5.2–21 mM, Sigma #856177). Most compounds were tested in 2-fold dilutions across the concentration range, while HGA and H_2_O_2_ were diluted 10-fold. All compounds were tested in shaken 96-well plates except for H_2_O_2_. To avoid volatile H_2_O_2_ from cross-reacting between conditions, 1-mL cultures were exposed to this compound in separate glass test tubes and culture in a roller drum.

### PI staining and quantification

During the HGA susceptibility assay, cell viability was determined by plating for CFUs and imaging propidium iodide-stained bacterial cells at each timepoint. To define cell boundaries, we used a KS79 *Lp* strain carrying the pON-GFP plasmid to constitutively express GFP (see “Strains and mutant construction” above). Cells at each timepoint were incubated with 2-µg/mL propidium iodide (Thermo fisher, #P1304MP) for 5 min in the dark just before imaging to stain permeable bacterial cells. To image the cells, stained cell samples were spotted onto GeneFrame agar pads (Fisher Scientific #AB-0576) and visualized on a Nikon TE2000 inverted scope using a ×60 oil immersion lens in brightfield, red (560 nm), and green (488 nm) channels. For each condition and time point, at least three fields of view were quantified, or a minimum of 60 cells each. The data presented here come from one of two independent experimental replicates.

Images were single blinded and manually counted for quantification. To determine cell count of green and red cells, the collected image was split into separate color channels using Fiji (47). Cells (defined as 0.1–10.0 μm^2^ round particles) were identified using the GFP signal. Cells were defined as PI+ if they appeared in both red and green channels or if they were visible only in the red channel.

### RNA-seq sample preparation and analysis

The samples prepared for RNA-seq were high- and low-density KS79 cells, with and without HGA. RNA was extracted after 1-h HGA exposure using the RNeasy Mini Kit (Qiagen #74104) as directed, with the addition of a bacterial lysis step using 50 μL 10 mM Tris-Cl, 1 mM EDTA, 1-mg/mL lysozyme prior to the addition of RLT buffer. RNA samples were treated with TURBO DNase (Fisher scientific #AM1907) to remove contaminating DNA. RNA quality was assessed using the Agilent Tapestation for concentration >50 ng/μL and RIN >6 prior to sequencing. Sequencing libraries were prepared using Illumina stranded RNA library preparation with RiboZero Plus rRNA depletion (Illumina #20040529).

Each library was sequenced with 50-bp paired-end reads, to a depth of ~12 M reads per sample. Raw sequencing reads were trimmed and filtered using trimmomatic (48). After trimming for quality, any reads less than 40 bp and average quality less than 30 were dropped. Trimmed filtered reads were mapped to the reference *Lp* genome (Genbank accession GCA_000008485.1) using kallisto (49). The mapped reads were analyzed for differential expression using the DESeq2 R package and a *P* value cutoff of 0.05 (50). DESeq2 normalized read counts across samples and performed the default B-H false discovery rate correction (51) on *P* values to produce the adjusted *P* values. We analyzed differential expression according to sample density (high vs low) and HGA (present or absent). Differentially expressed genes were defined as genes with >2-fold change in expression and an adjusted *P* value <0.05. To validate that experimental conditions correlated well within replicate, normalized count data were plotted on a scatter matrix using the pairs R function. To assess variability in our data, we used the vst and plotPCA functions of DESeq2. The differential expression of candidate differentially expressed genes (DEGs) and pathways of interest were displayed in heatmaps using ggplot2 v.3.3.3 (52). *Legionella* genes involved in iron acquisition and HGA production were defined based on Cianciotto (26). Genes involved in ROS response were based on references 12 and 53 – 55. Genes involved in the *Legionella* quorum sensing pathway were based on references 20 and 56, and those in the flagellar regulon were based on Albert-Weissenberger et al. (28).

### Preparation of low- and high-density *Lp* for Amplex Red assay

For the experiments in Fig. 4B and C, we used a *hisC2*:Tn *Lp* strain from reference 8 that does not secrete HGA to ensure that only exogenous HGA was present. For Fig. 4B, high- and low-density *Lp* bacteria were washed in PBS and mixed with 125-µM HGA, 50 µM H_2_O_2_ solution, or PBS alone as a control. Each condition was incubated for 4 h at 37°C in a roller drum. After 4 h, each condition was sampled for Amplex Red assays (see “ROS measurements via Amplex Red assay” below). In Fig. 4C, *Lp* bacteria were resuspended in PBS to a density of ~5e9 CFU/mL and then were diluted in serial, twofold dilutions down to a density of 3.9e7 CFU/mL. Cultures at each density were then treated with H_2_O_2_ at a final concentration of 50 µM for 5 min. Finally, experimental tubes were briefly vortexed and sampled for Amplex Red readings. Samples were read in parallel to an H_2_O_2_ standard curve to estimate the quantity of hydroperoxides present in experimental samples.

### Preparation and activity of *L. pneumophila* supernatants

Supernatants from high-density cells (HDsup) were prepared by washing stationary phase *L. pneumophila* in PBS and incubating 1.5 mL of 4–9E9 CFU/mL cells in a rolling-drum incubator at 37°C for 1 h. Supernatants were harvested by centrifugation followed by filter sterilization with a 0.22-µm filter. To test if HDsup could quench HGA-derived ROS or H_2_O_2_, we first incubated 125 µM of HGA or 50 µM of H_2_O_2_ in PBS at 37°C for 4 h in a rolling drum incubator to fully generate ROS. We then mixed 150 μL of the preoxidized HGA or H_2_O_2_ with 2.85 mL of HDsup or PBS. The 50 µM H_2_O_2_ samples were compared with 50 µM final in HDsup (i.e., 150 μL of 1 mM H_2_O_2_ + 2.85-mL HDsup). All conditions were briefly vortexed and then sampled for Amplex Red readings after 5-min co-incubation.

To test if supernatants impacted bacterial susceptibility to HGA, 1.5 mL of *L. pneumophila* cells (at high or low density) were added to 1.35 mL HDsup and 150 μL of HGA (final HGA concentration 125 µM).

### ROS measurements via Amplex Red assay

To quantify the hydroperoxides present in conditions treated with HGA, H_2_O_2_, or *L. pneumophila* bacteria, we used the reagent Amplex Red (Thermo Fisher #A12222), which generates a fluorescent compound in the presence of hydroperoxides. Amplex Red solution consisted of 50 μM of Amplex Red and 0.1 U of horseradish peroxidase (Sigma #77332), which was pre-incubated for 10 min at 37°C. Then, 100 μL of Amplex reaction solution was added to 20 μL of experimental culture in an opaque 96-well plate. Fluorescence was measured after 5 min with a Agilent Biotek Cytation 5 plate reader at 590 nm using an excitation wavelength of 530 nm. Fluorescence values were converted to H_2_O_2_ concentrations using a standard curve of known H_2_O_2_ concentrations in parallel.

## Data Availability

Data have been deposited as follows: RNA-seq read archive (BioProject ID), PRJNA943215; RNA-seq read processing and analysis scripts, https://github.com/mliannholland/hga_rnaseq

## SUPPLEMENTAL MATERIAL

Fig. S1 mbio.01207-23-s0001.tif HGA-mediated killing depends on culture volume and shaking conditions.

Fig. S2 mbio.01207-23-s0002.tif
*L. pneumophila*'s density-dependent susceptibility to HGA and H_2_O_2_ does not extend to other oxidative stressors nor to other bacterial species.

Fig. S3 mbio.01207-23-s0003.tif
*L. pneumophila*'s density-dependent susceptibility to HGA does not depend on the HGA synthesis or Lqs quorum sensing pathways.

Fig. S4 mbio.01207-23-s0004.pdf Validation of RNA-seq analyses.

Supplemental Legends mbio.01207-23-s0005.docx Legends for four supplemental figures and one supplemental table.

Table S1 mbio.01207-23-s0006.xlsx RNA-seq normalized read counts and statistical analyses comparing expression between high- and low-density *Legionella* cultures with and without HGA, after 1 hour of incubation.

ASM does not own the copyrights to Supplemental Material that may be linked to, or accessed through, an article. The authors have granted ASM a non-exclusive, world-wide license to publish the Supplemental Material files. Please contact the corresponding author directly for reuse.
