# Supplementary figures and images for: L. pneumophila resists its self-harming metabolite HGA via secreted factors and collective peroxide scavenging
Source: mBio. 2023 Sep 20;14(5):e01207-23. doi: 10.1128/mbio.01207-23 (PMC10653783; doi:10.1128/mbio.01207-23)

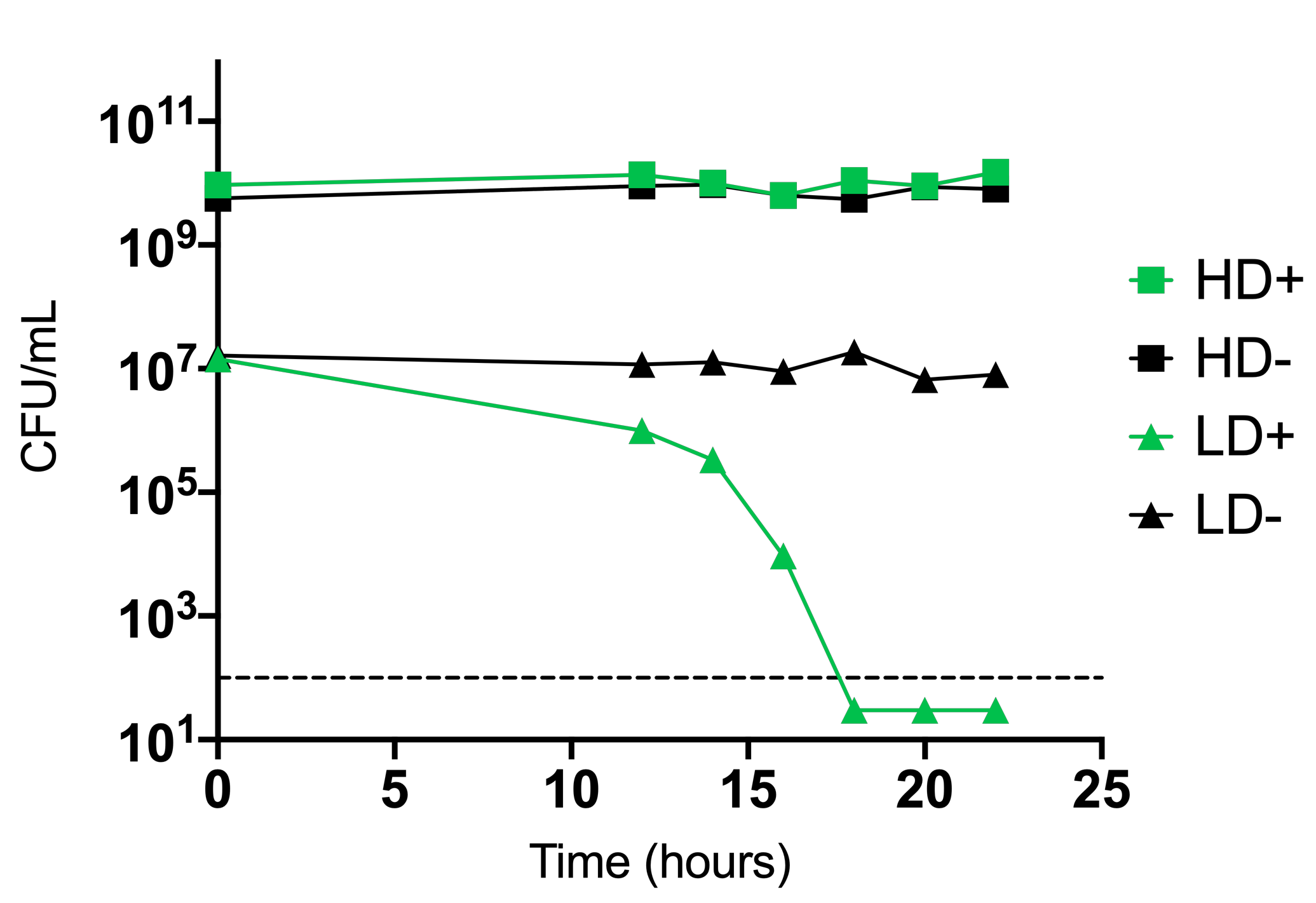

Supplement: Fig. S1 — HGA-mediated killing depends on culture volume and shaking conditions. [file mbio.01207-23-s0001.tif]

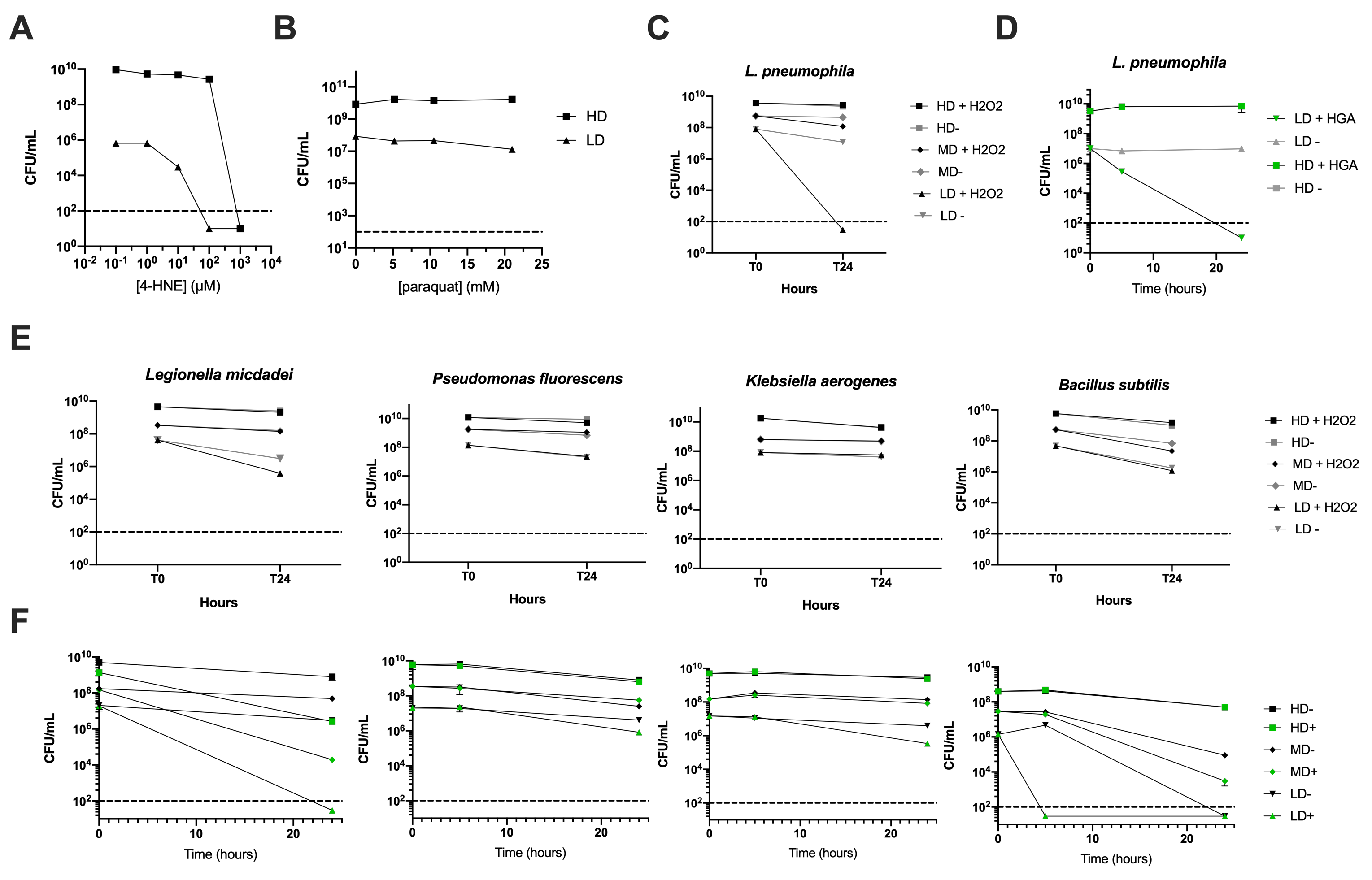

Supplement: Fig. S2 — L. pneumophila's density-dependent susceptibility to HGA and H2O2 does not extend to other oxidative stressors nor to other bacterial species. [file mbio.01207-23-s0002.tif]

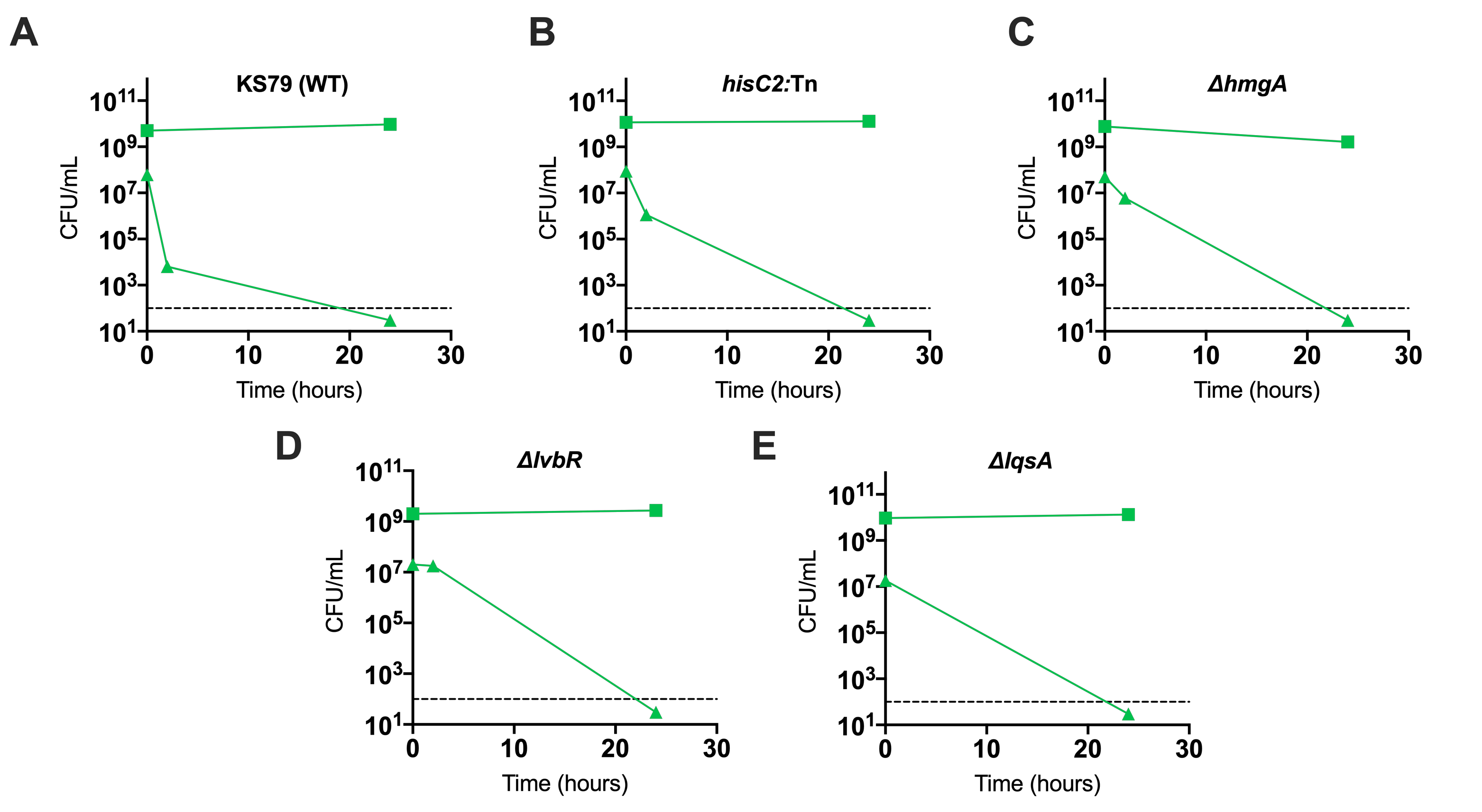

Supplement: Fig. S3 — L. pneumophila's density-dependent susceptibility to HGA does not depend on the HGA synthesis or Lqs quorum sensing pathways. [file mbio.01207-23-s0003.tif]

**A**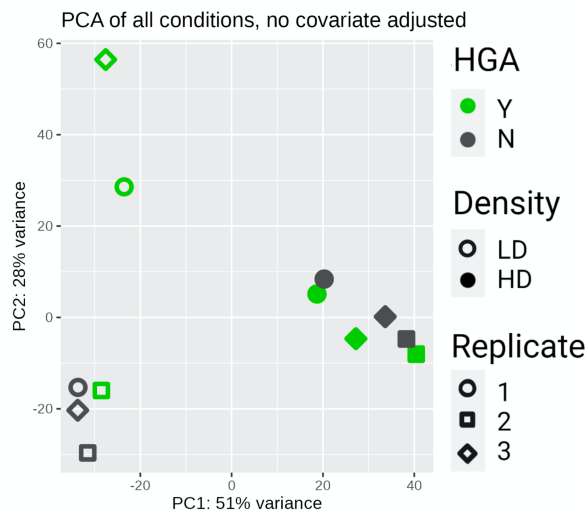**B**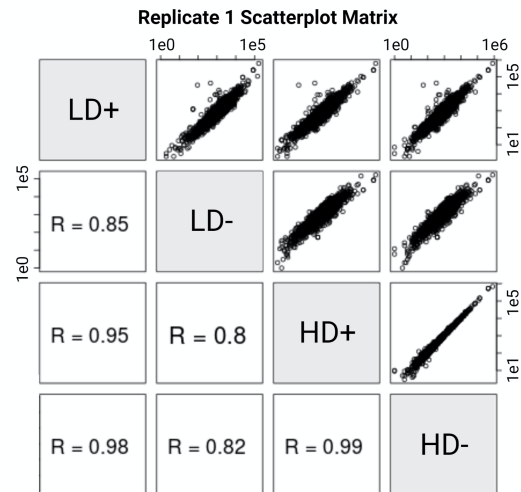**C**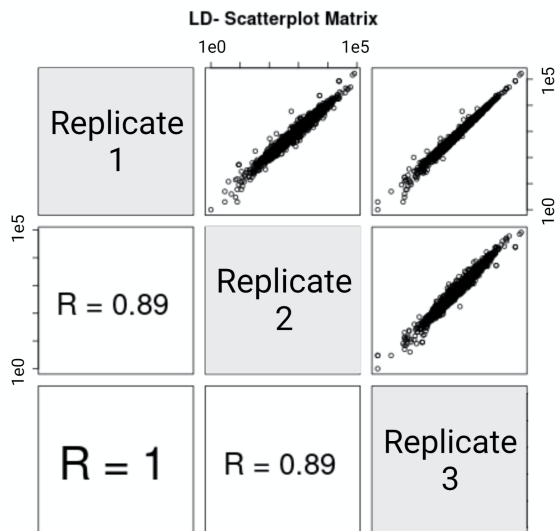**D**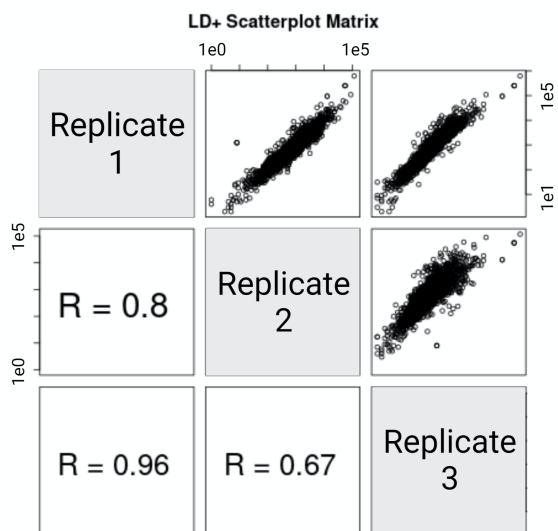**E**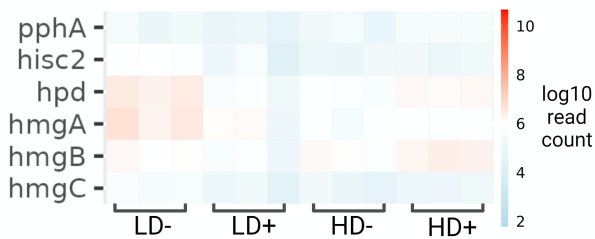**F**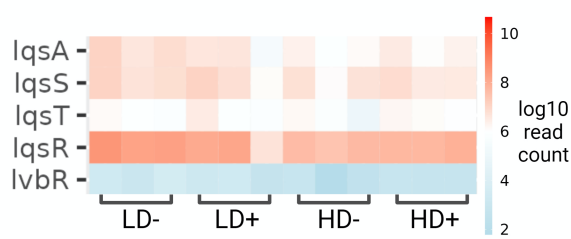

Supplement: Fig. S4 — Validation of RNA-seq analyses. [file mbio.01207-23-s0004.pdf]
