# Supplementary material for: L. pneumophila resists its self-harming metabolite HGA via secreted factors and collective peroxide scavenging
Source: mBio. 2023 Sep 20;14(5):e01207-23. doi: 10.1128/mbio.01207-23 (PMC10653783; doi:10.1128/mbio.01207-23)
Supplement: Supplemental Legends — Legends for four supplemental figures and one supplemental table. [file mbio.01207-23-s0005.docx]

**Supplemental Figure Legends:**

**Supplemental Figure 1.** HGA-mediated killing depends on culture volume and shaking conditions. Low-density cells exposed to HGA (LD+ cells) die after 16-18 hours when in 100uL volumes in a 96-well plate, shown here. This is considerably slower than HGA-mediated killing in 3mL culture tubes within a roller drum (Figure 1B).

**Supplemental Figure 2.** *L. pneumophila’s* density-dependent susceptibility to HGA and H2O2 does not extend to other oxidative stressors nor to other bacterial species. **A)** Similar concentrations of 4-HNE (<10X different) are toxic to high- and low-density *L. pneumophila*. **B)** In PBS, *L. pneumophila* is not susceptible to paraquat. **C)** *L. pneumophila* susceptibility to 300 µM H2O2 (compare to E). All other species tested showed minimal sensitivity to H2O2. **D)** *L. pneumophila* susceptibility to 125 µM HGA (compare to F). **E-F)** Susceptibility of other bacterial species at high, medium, or low density (HD, MD, LD) to H2O2 **(E)** or HGA **(F)**. *L. micdadei* exhibited some susceptibility to HGA at all cell densities. *B. subtilis* had HGA susceptibility at low densities in addition to poor overall viability in PBS.

**Supplemental Figure 3.** *L. pneumophila’s* density-dependent susceptibility to HGA does not depend on the HGA synthesis or Lqs quorum sensing pathways. **A)** Wild-type *L. pneumophila* susceptibility to 125 µM HGA between high-density (square) or low-density (triangle) cultures. **B-D)** HGA susceptibility is unaffected by mutations that eliminate **(B)** or enhance **(C)** HGA secretion, nor by the deletion of genes encoding the Lqs-regulated transcription factor *lvbR* **(D)** or the Lqs autoinducer synthase *lqsA* **(E).**

**Supplemental Figure 4.** Validation of RNA-seq analyses. **A)** Principal component analysis of all RNA-seq samples according to HGA exposure (color), cell density (fill), and replicate (shape). **B-D)** Scatter matrices comparing each gene’s normalized counts within replicate 1 **(B)**, between replicates of low density cells without HGA **(C)**, and between replicates of low density cells with HGA **(D)**. **E-F)** Heatmaps showing that neither the HGA biosynthesis pathway **(E)** nor the Legionella quorum sensing (Lqs) pathway **(F)** are differentially expressed in these conditions.

**Supplemental Table 1.**

RNA-seq normalized read counts and statistical analyses comparing expression between high- and low-density (HD and LD) Legionella cultures with and without HGA (withHGA and noHGA), after 1 hour incubation.
